# Supplementary material for: Fetal Renal Duplicated Collecting System at 14–16 Weeks of Gestation
Source: J Clin Med. 2023 Nov 16;12(22):7124. doi: 10.3390/jcm12227124 (PMC10672134; doi:10.3390/jcm12227124)
Supplement: Supplementary file 1 [file jcm-12-07124-s001.zip › jcm-2579555-supplementary.pptx]

## Slide 1
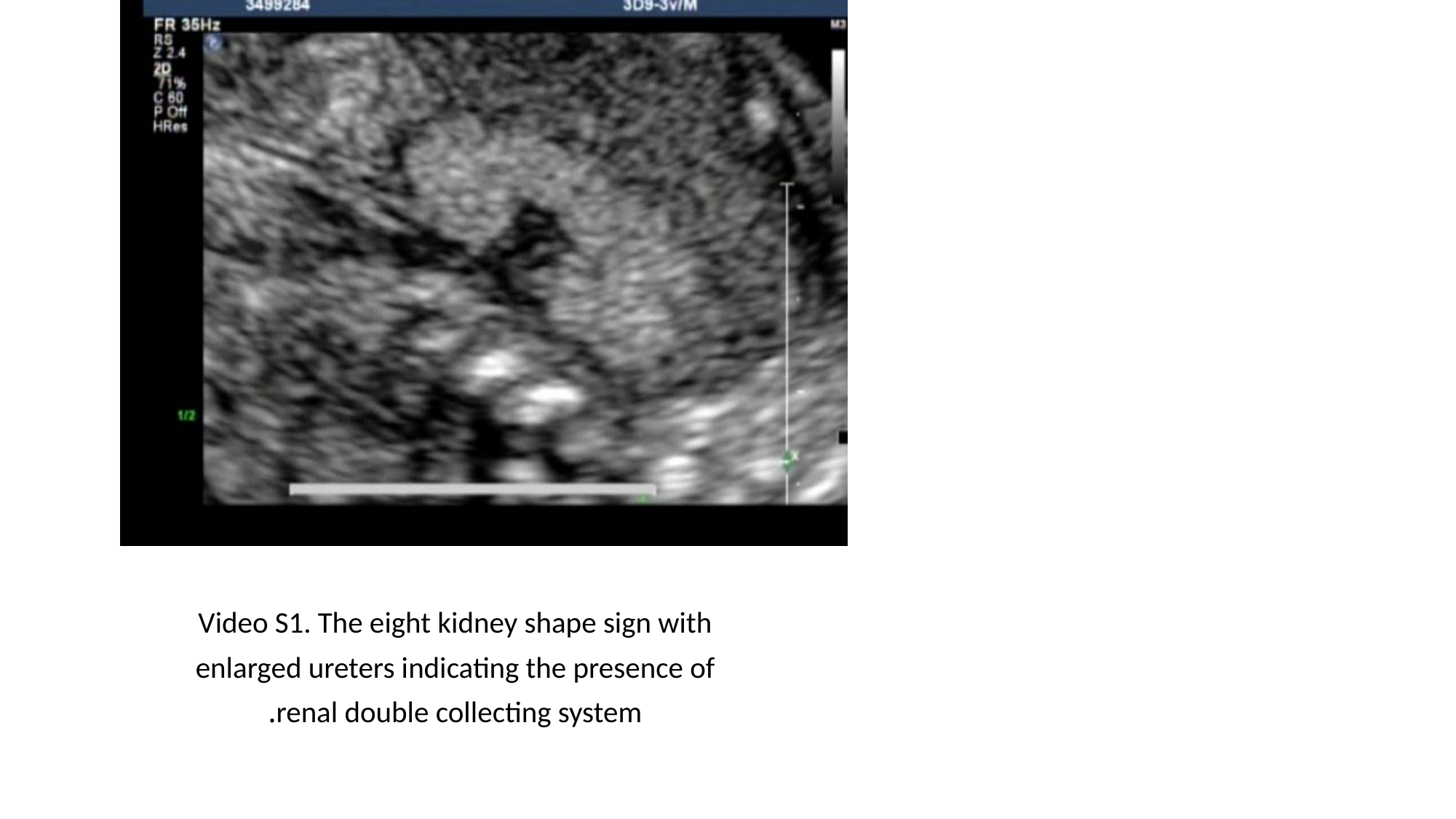

#
Video S1. The eight kidney shape sign with
enlarged ureters indicating the presence of
renal double collecting system.
